# Effects of Malnutrition on Left Ventricular Mass in a North-Malagasy Children Population

**DOI:** 10.1371/journal.pone.0154523

**Published:** 2016-05-03

**Authors:** Giuseppe Di Gioia, Antonio Creta, Mario Fittipaldi, Riccardo Giorgino, Fabio Quintarelli, Umberto Satriano, Alessandro Cruciani, Vincenzo Antinolfi, Stefano Di Berardino, Davide Costanzo, Ranieri Bettini, Giuseppe Mangiameli, Marco Caricato, Giovanni Mottini

**Affiliations:** 1 Department of Medicine and Surgery, Cardiology Unit, Università Campus Bio-Medico di Roma, Rome, Italy; 2 Paediatric Cardiothoracic Surgery, Starship Greenlane Paediatric and Congenital Heart Service, Auckland, New Zealand; 3 Department of Medicine and Surgery, Pediatrics Service, Università Campus Bio-Medico di Roma, Rome, Italy; 4 Department of Heart and Great Vessels “*Attilio Reale*”, Sapienza University, Rome, Italy; 5 Cardiology Department, University of Pisa, Pisa, Italy; 6 Department of Medicine and Surgery, Unit of Geriatric Surgery, Università Campus Bio-Medico di Roma, Rome, Italy; 7 Department of Medicine and Surgery, Institute of Philosophy of Scientific and Technological Practice (FAST), Università Campus Bio-Medico di Roma, Rome, Italy; National Institute of Health, ITALY

## Abstract

**Background:**

Malnutrition among children population of less developed countries is a major health problem. Inadequate food intake and infectious diseases are combined to increase further the prevalence. Malnourishment brings to muscle cells loss with development of cardiac complications, like arrhythmias, cardiomyopathy and sudden death. In developed countries, malnutrition has generally a different etiology, like chronic diseases. The aim of our study was to investigate the correlation between malnutrition and left ventricular mass in an African children population.

**Methods:**

313 children were studied, in the region of Antsiranana, Madagascar, with age ranging from 4 to 16 years old (mean 7,8 ± 3 years). A clinical and echocardiographic evaluation was performed with annotation of anthropometric and left ventricle parameters. Malnutrition was defined as a body mass index (BMI) value age- and sex-specific of 16, 17 and 18,5 at the age of 18, or under the 15^th^ percentile. Left ventricle mass was indexed by height^2.7^ (LVMI).

**Results:**

We identified a very high prevalence of children malnutrition: 124 children, according to BMI values, and 100 children under the 15^th^ percentile. LVMI values have shown to be increased in proportion to BMI percentiles ranging from 29,8 ± 10,8 g/m^2.7^ in the malnutrition group to 45 ± 15,1 g/m^2.7^ in >95^th^ percentile group. LVMI values in children < 15^th^ BMI percentile were significantly lower compared to normal nutritional status (29,8 ± 10,8 g/m^2,7^ vs. 32,9 ± 12,1 g/m^2,7^, p = 0.02). Also with BMI values evaluation, malnourished children showed statistically lower values of LVMI (29,3 ± 10,1 g/m^2,7^ vs. 33,6 ± 12,5 g/m^2,7^, p = 0.001).

**Conclusion:**

In African children population, the malnourishment status is correlated with cardiac muscle mass decrease, which appears to be reduced in proportion to the decrease in body size.

## Introduction

Children malnourishment is a common disease in less-developed countries mostly due to inadequate food intake, but also political or socioeconomic factors affecting life quality of the population. It constitutes a major public health problem in the developing world and represents the most important risk factor for the burden of disease. About 5 million children have died worldwide directly or indirectly due to malnutrition and 9 children/minute die as a consequence of malnutrition; World Health Organization (WHO) has identified childhood malnutrition as the most lethal form of malnutrition [1,2]. Globally, it is estimated that there are about 20 million children who are severely malnourished, most of them live in south Asia and in sub-Saharan Africa [3]. In these communities the high prevalence of poor diet and infectious diseases regularly gives rise to a vicious cycle [4]. Some chronic clinical conditions, like renal diseases, cystic fibrosis and acquired immunodeficiency syndrome or social problems like parental drug addiction or alcoholism, can bring to a deficient nutritional status also in advanced countries [5,6]. Malnourished children suffer several alterations in body composition, together with loss of skeletal and cardiac muscle mass, complicated by electrolyte imbalance, mineral and vitamin deficiencies that could bring also to cardiac abnormalities, including hypotension, cardiac arrhythmias, cardiomyopathy, cardiac failure and even sudden death [7,8]. A consensus of expert highlighted muscle mass wasting as the key feature of disease-associated malnutrition [9]. Malnutrition affecting left ventricle mass was previously studied in Western countries, but no data exist regarding less developed countries where malnourishment’s etiology is different. One of the most commonly used and recommended parameters to assess nutritional status in adults is the body mass index (BMI). An adaptation for children has been limited in the past, because BMI is not constant across the pediatric age range [10]. In recent years, several countries added age- and sex-specific BMI values and BMI percentiles to their growth charts. BMI percentile provides a way of comparing a child’s weight, adjusted for height, with a reference group of the same age, but not necessarily the same stature.

The aim of our study was to evaluate the correlation between nutritional status and left ventricle mass in an African children population.

## Material and Methods

We performed a clinical and echocardiographic evaluation of 313 consecutive African children up to 16 years of age. This study was performed during a medical work camp, directed by Campus Bio-Medico University of Rome, with the participation of a team composed by cardiologists, pediatricians, surgeons and medical students. Having our main base (also headquarter) into the “Clinique Médico-Surgicale St. Damien” of Ambanja, we investigated the region of Antsiranana, in the north of Madagascar, visiting the catholic schools of Sekoly Venance Manifatra “SE.VE.MA.” and Foyer Mangafaly in Ambanja and the college Sainte Therese de l’Enfant Jesus in Maromandia, during the month of October 2015. In primary and high schools we randomly selected a class from each group of age to enroll individuals from 4 to 16 years of age.

At the “Clinique St. Damien” we enrolled children who came for routine visits or screening, or in-patients. All individuals underwent 3 steps. The first one was registration into our database with name, surname (when available), date-of-birth and demographical parameters. The second step included the clinical evaluation, it was performed by a pediatrician, with annotation of weight, height, subsequent body mass index (BMI) calculation, blood pressure, heart rate and cardiac auscultation. The clinical questionnaire was undertaken by a visiting medical student—under the supervision of a medical doctor—aided, when necessary, by a local interpreter who had been educated in advance. Teachers or their co-workers helped the younger children understanding and answering the questions. Finally, each child was submitted to a transthoracic echocardiogram, performed by two expert cardiologists, with a portable Esaote Mylab Five ecograph (Esaote s.p.a., Italia), equipped with a high-quality S5-1 transducer probe. Subjects were studied in the left lateral recumbent position and all standard echocardiographic views were acquired. Measurements of LV dimensions and volumes were obtained according to current American Society of Echocardiography (ASE) recommendations [11]. The LV inner dimensions were measured at end-diastole and end-systole using M-Mode echocardiography in the parasternal long axis view. End-diastole was defined as the frame following mitral valve closure, end-systole as the frame following mitral valve opening. Modified Simpson‘s biplane method by biapical views was used to calculate LV volumes and ejection fraction (EF). Preserved LV function was defined as ≥ 60%. Left ventricle mass (LVM) was calculated based on Devereux’s formula [12] and indexed by height^2.7^ [13] (LVMI). In particular, LVM results from the formula: LVM = ¼ 0.8 x (1.04 [LVIDd + PWTd + SWTd)^3^ –(LVIDd)^3^]) + 0.6 g, where LVIDd is the left ventricular internal dimension at the end-diastole, PWTd is the posterior wall thickness at the end-diastole, and SWTd is the septal wall thickness at the end-diastole [11]. LVH was defined as a LVMI value > 51 g/height^2.7^ or above the 95^th^ percentile age- and sex-specific [14]. BMI values to define malnutrition in children were adapted according to age and sex [15] and thinness grade 1, 2 and 3 (also called mild, moderate and severe malnutrition) were defined as the value to pass through a BMI of 16, 17 and 18,5 at the age of 18. Regarding BMI percentiles, the malnutrition status was diagnosed if a child was found to be under the 15^th^ percentile. The BMI 15^th^ percentile as cut-off value for malnutrition has been previously validated by studies that used DEXA scan and average skinfold measurements [16]. Mathematically, the 15th percentile corresponds approximately to the -1 standard deviation in a normalized distribution. Since widespread population illiteracy, written informed consent was difficult to obtain. So, a verbal informed consent, with teacher’s help to translate into local language, was obtained from children’s parents, who gave study approval to school’s teachers and our research group. The study was reviewed and approved—before it began—by ethics committees of local hospital “Clinique Médico-Surgicale St. Damien” of Ambanja and University Campus Bio-Medico of Rome.

### Statistical analysis

Categorical variables are expressed as frequencies and percentages in parenthesis, and are compared using Fisher’s exact test or Chi-square test, as appropriate. Normality criteria were checked and met for any continuous variable, which are presented as mean and standard deviation and compared using Student t-test for independent data. Correlation between continuous variables was calculated using Pearson’s test. A P value less than 0.05 was considered statistically significant. Statistical analysis was performed with STATA Statistics for Windows (SE, version 13).

## Results

313 children of African race were studied, with a slight prevalence of female sex (53%). Mean age was 7,8 ± 3 years, ranging from 4 to 16 years of age. Clinical and echocardiographic parameters of the total study population are listed in Table 1. All children had normal arterial blood pressure. At echocardiographic evaluation, a valve disease was diagnosed in 4 children (3 of them having moderate mitral regurgitation and one aortic regurgitation). Two mitral valve prolapses with mild regurgitation were individuated. Five congenital heart defect (1,6%) were diagnosed: two inter-atrial septum defect, one inter-ventricular septum defect, one boy with bicuspid aortic valve and one four-year-old girl affected by cor triatriatum sinister.

**Table 1:** Characteristics of study population.

N° of children	313
Male, n (%)	146 (47)
Age, years	7,8 ± 3
Height, cm	120 ± 20
Weight, kg	23,2 ± 8,2
BMI, kg/m^2^	15,5 ± 1,9
BSA	0,6 ± 0,3
SP, mmHg	113 ± 8
DP, mmHg	70 ± 7
HR, bpm	103 ± 18
LVEDD, mm	35 ± 4
IVS, mm	6,3 ± 1,3
PW, mm	5,3 ± 0,9
LVEF, (%)	64,1
LVM, g	50,5 ± 16
LVMI, g/m^2.7^	31,9 ± 11,8

Demographical, clinical and echocardiographic parameters of general population. The numbers are expressed as numerical values (%) or mean ± standard deviation.

Abbreviations. BMI: body mass index; BSA: body surface area; DP: diastolic pressure; HR: heart rate; IVS: inter-ventricular septum; LVEDD: left ventricle end-diastolic diameter; LVEF: left ventricle ejection fraction; LVM: left ventricle mass; LVMI: left ventricle mass indexed; PW: posterior wall; SP: systolic pressure.

**Fig 1:**
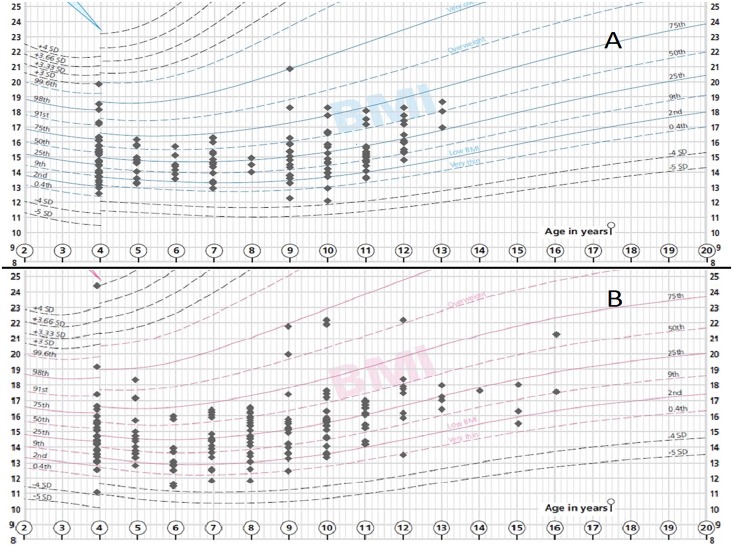
Children distribution according to BMI percentiles. BMI percentile distribution of our children population according to age and sex. **A**: male sex. **B**: female sex. *(Adapted from Royal College of Paediatrics and Child Health BMI charts)*.

In our study children population, after adjusting BMI values for age and sex [15], we observed a high percentage of malnutrition status (124 children, 40%) with a severe malnutrition identified in 13 (4%) children; moderate in 41 (13%) and mild in 70 (22%). Dividing the children population according to BMI percentiles (Fig 1), we observed that more than 85% of children were under 50^th^ percentile. In the detail, 100 children (32%) were under 15^th^ percentile, indicating a pathological nutritional status; 113 children (36%) were between the 15^th^ and 25^th^; 61 (19%) between 25^th^ and 50^th^; 21 children (7%) between 50^th^ and 75^th^; 8 (3%) between 75^th^ and 95th and only 10 (3%) over 95^th^ percentile.

**Fig 2:**
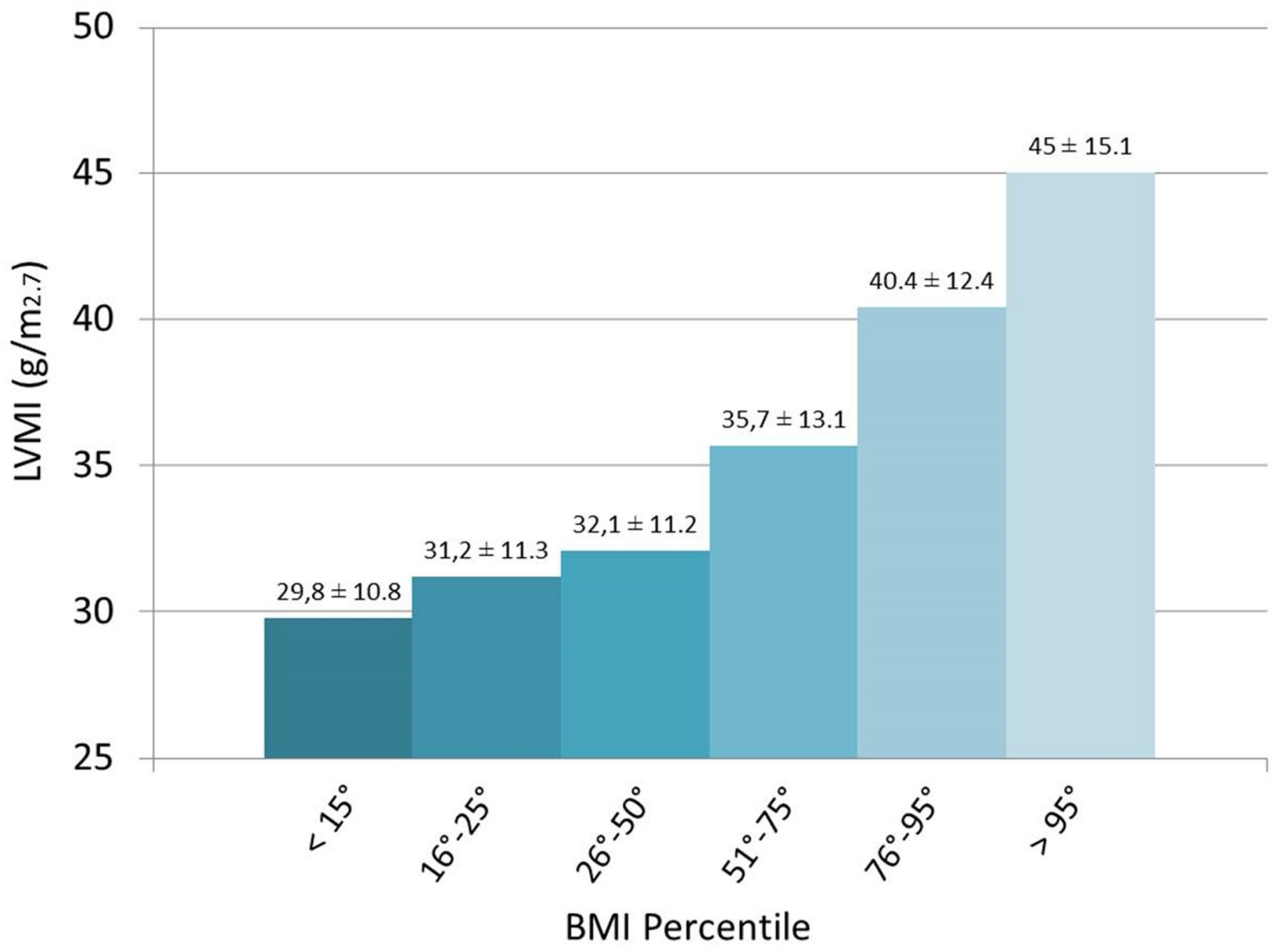
Correlation between LVMI and BMI percentiles. LVMI values decrease in proportion to the reduction of BMI percentiles.

LVMI values were strictly correlated to the BMI percentiles ranging from 29,8 ± 10,8 g/m^2.7^ in the malnutrition group (< 15^th^ percentile) to LVMI of 45 ± 15,1 g/m^2.7^ in >95^th^ percentile group (see Fig 2). Children with a lower BMI, according to their age and sex, entering in a malnourishment status, had statistically lower value of LVMI compared to children with normal nutrition profile (29,3 ± 10,1 g/m^2.7^ vs. 33,6 ± 12,5 g/m^2.7^ respectively, p = 0.001) (Table 2). Also dividing the population according to BMI percentiles, the same results were obtained: LVMI values in children < 15^th^ percentile were statistically lower, compared to normal nutritional status (29,8 ± 10,8 g/m^2.7^ vs. 32,9 ± 12,1 g/m^2.7^ respectively, p = 0.02).

**Table 2:** Correlation between malnutrition state (according to BMI and BMI percentiles) and LVMI.

	BMI			BMI percentile		
	Mal-nourished	Normal nutrition	p	Mal-nourished	Normal nutrition	p
Children, n (%)	124 (40)	189 (60)		100 (41)	213 (59)	
LVMI (g/m^2.7^)	29,3 ± 10,1	33,6 ± 12,5	0.001	29,8 ± 10,8	32,9 ± 12,1	0.02

Mal-nourished children showed statistically significant lower values of LVMI. The numbers are expressed as numerical values (%) or mean ± standard deviation.

Abbreviations. BMI: body mass index; LVMI: left ventricle mass indexed.

Malnourished children were more frequently male (63/146, 43%), among which 3 children suffering of severe, 22 of moderate and 38 of mild malnutrition. Regarding females, malnutrition was diagnosed in 61 of 167 (37%), with 10 children affected by severe, 19 by moderate and 32 by mild malnutrition state. Between malnourished and normal nutrition group (Table 3) there were no differences regarding sex prevalence, age, height, BSA, arterial blood pressure and heart rate, while there was a statistically difference on echocardiographic parameters. Malnourished children showed a smaller LV dimension (34,4 ± 3,5 mm vs. 35,9 ± 3,9 mm; p = 0.006) a thinner IVS (5,9 ± 1,1 mm vs. 6,5 ± 1,3 mm; p<0.001) and PW (5,1 ± 0,8 mm vs. 5,3 ± 0,8 mm; p = 0.03) and lower values of LVM (45,8 ± 14,4 g vs. 53,6 ± 16,3 g; p<0.001) and LVMI (29,3 ± 10,1 g/m^2.7^ vs. 33,6 ± 12,5 g/m^2.7^; p = 0.001). Although this differences in LV dimensions, LVEF—that is the most used parameter for the evaluation of ventricular systolic function—showed no significant change in the malnourished group compared to normal weight children (p = 0.107).

**Table 3:** Normal nutrition status versus mal-nourished children (according to BMI).

	Normal nutrition (n = 189)	Mal-nourished (n = 124)	P
Male, n (%)	83 (44)	63 (51)	0.23
Age, years	7,9 ± 2,9	7,5 ± 2,9	0.23
BMI (kg/m^2^)	16,5 ± 1,7	14 ± 1	0.001
Height, m	1,21 ± 0,17	1,19 ± 0,16	0.29
Weight, kg	24,9 ± 8,6	20,4 ± 6,6	<0.001
BSA	0,6 ± 0,34	0,59 ± 0,27	0.78
SP, mmHg	113 ± 8	114 ± 8	0.28
DP, mmHg	69 ± 7	70 ± 7	0.21
HR, bpm	103 ± 17	102 ± 19	0.62
LVEF	64,3 ± 2,1	63,9 ± 2,2	0.10
LVEDD, mm	35,9 ± 3,9	34,4 ± 3,5	0.006
IVS, mm	6,5 ± 1,3	5,9 ± 1,1	<0.001
PW, mm	5,3 ± 0,8	5,1 ± 0,8	0.03
LVM, g	53,6 ± 16,3	45,8 ± 14,4	<0.001
LVMI, g/m^2.7^	33,6 ± 12,5	29,3 ± 10,1	0.001

Comparison of demographical, clinical and echocardiographic data between mal-nourished and normal nutrition status children according to BMI. Mal-nourished children showed statistically lower values of all echocardiographic parameters, except of LVEF. The numbers are expressed as numerical values (%) or mean ± standard deviation.

Abbreviations. BMI: body mass index; BSA: body surface area; DP: diastolic pressure; HR: heart rate; IVS: inter-ventricular septum; LVEDD: left ventricle end-diastolic diameter; LVEF: left ventricle ejection fraction; LVM: left ventricle mass; LVMI: left ventricle mass indexed; PW: posterior wall; SP: systolic pressure.

**Fig 3:**
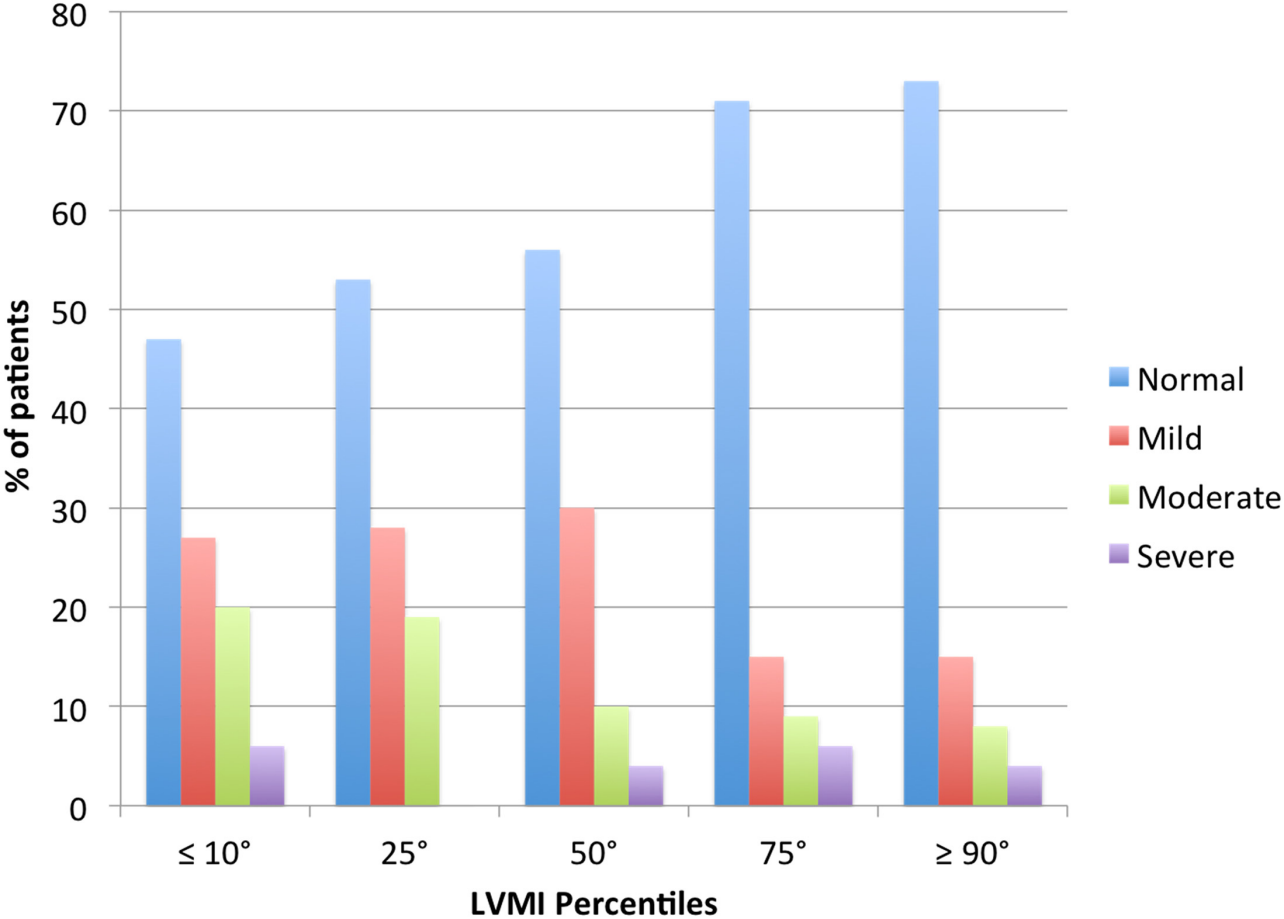
Nutritional status and LVMI percentiles. Nutritional children status divided as normal or mild, moderate or severe mal-nourishment according to LVMI Percentiles. Number of children with normal nutritional status was higher in greater LVMI percentiles.

According to the work of Khoury et al. [17] which gave age and sex-specific reference values for children’s LVMI, our study population was divided as follows: 86 children (27%) were in the ≤10^th^ percentile, 32 (10%) were in the 25^th^, 57 (18%) in the 50^th^; 55 (18%) in the 75^th^, 30 (10%) in the 90^th^, 13 children (4%) in the 95^th^ and 40 (13%) with values above 95^th^ percentile. 24 children (8%) had a LVM value > 51 g/m^2.7^ (ranging from 52,3 to 82,3 g/m^2.7^), indicating LVH. LVMI percentiles were related to BMI. As shown in Fig 3, children with normal nutritional status increased progressively with the growth of LVMI percentiles (from 47% in children with LVMI ≤ 10° percentile to 73% in those with LVMI ≥ 90° percentile).

## Discussion

In less-developed countries, malnutrition is major health problem affecting most of the population, both children and adults. According to WHO 2008–2012 data, malnutrition prevalence in Madagascar is alarming, affecting 36% of children population, presenting in a moderate to severe underweight status. In our study we found a malnutrition status in 40% of children population, according to BMI values, and 32% of children under 15^th^ percentile. Poor health literacy and misconceptions regarding proper nutrition and malnutrition, and insufficient production of adequate food products are at the base of this problem. Nutritional status and heart disease are strictly connected with an important influence on long-term prognosis. Children affected by malnutrition can exhibit a series of cardiovascular abnormalities, including hypotension, cardiac arrhythmias, cardiomyopathy, cardiac failure and in some cases, sudden death [18–21]. Nowadays, pathogenic mechanisms of morphological and functional cardiac alterations associated with malnutrition have not been still completely elucidated. Necropsy studies have demonstrated myocardial degeneration, myocytolysis, fat infiltration or substitution of muscle cells by fat (adipose) tissue. Membrane action potential of myocardic (myocardial or cardiac muscle) cells could be altered in malnourished subjects due to structural muscular changes, variations of blood flow and water content or potassium channel modification observed in hypotrophic cardiomyocytes [22]. Previous old echocardiographic studies showed a correlation between protein energy malnutrition (PEM) and left ventricle abnormalities. Anyway, there are several consistent differences between previous studies and ours. In fact, the work of Bergman et al. [23], was based on only 21 subjects and it was evaluated only the correlation with cardiac dimension, without any study on left ventricular mass. Singh et colleagues [24] studied 46 malnourished children in a different range of age (between 3–48 months), while the study by Kothari et al. [25] was about only 25 malnourished children, also investigating a range of age different from our study (one to five years old children). Moreover, all this studies were conducted in developed countries. More recently, Olivers et al. found that the decrement of LVM and LVMI was proportional to the decrement in the total body mass [7]. Also the study by Faddan et al., found that in children with PEM, echocardiographic evaluation revealed a significantly lower IVS thickness, PW thickness and LVM than the control group [26]. In our study, we found that children affected by any degree of malnutrition had lower value of LVMI compared to normal nutritional status children. Moreover, LVMI increased in proportion to the increase of BMI also in children with a normal nutritional status. Our findings are concordant with those found in previous studies, which reported that in patients with malnutrition the heart is not capable to escape from atrophy affecting other organs [8,27]. Cunha et al. [28] suggested that the cause of diminished cardiac mass in adult patients with PEM was slow myocardial anabolic rate rather than increased catabolism.

Scientific controversy exists whether malnourished children’s heart has normal or reduced systolic LV function. [23,24,29]. The study by Öcal et al. [8] and Bergman et al. [23] showed that both LV and fractional shortening were not different in the group with PEM compared to control group. On the other side, Phornphatkul et al. [20] demonstrated that children with PEM had associated cardiac muscle wasting and LV dysfunction and, moreover, systolic function was further reduced with a loss in bodyweight of more than 40% [24]. In our study, even if LV mass is reduced in proportion to the decrease of BMI, LVEF was not abnormal. This result shows that LV systolic function is preserved despite the loss of myocardial mass.

An interesting data emerged from our study was the high prevalence of children showing LVH, revealed both from the value of LVMI—adjusted by age and sex (24 children, 8%)–and from LVMI > 95^th^ percentile (40 children, 13%). In fact, the prevalence of LVH in pediatric hypertensive population has been reported to vary from 8 to 41% depending on the criteria used for determining hypertension and LVMI [30]. Our study population was not affected by arterial hypertension and showed mean lower BMI values than developed countries, so that, seen the strictly connection between BMI and LVMI, we expected a lower percentage of LVH. Moreover, 4 children showing LVH had also a malnutrition status. We don’t know if in these children there was any factor preventing heart muscle cells from wasting during malnutrition, or if there is a concomitant heart comorbidity affecting cardiac mass. Our study is the first to highlight the correlation between malnutrition and cardiac mass in a less-developed country children population, where the main cause of malnutrition is poor diet intake.

## Conclusion

Our study showed that, in an African children population, a mal-nourishment status is correlated with cardiac muscle mass decrease that appears to be reduced in proportion to the decrease in body size, evaluated through BMI and BMI percentiles.
